# Recent transmission of dengue virus and associated risk Facors among residents of Kassala state, eastern Sudan

**DOI:** 10.1186/s12889-020-08656-y

**Published:** 2020-04-19

**Authors:** Mawahib H. Eldigail, Hazem A. Abubaker, Fatima A. Khalid, Tajeldin M. Abdallah, Ibrahim A. Adam, Gamal K. Adam, Rabie A. Babiker, Mohamed E. Ahmed, Eltahir M. Haroun, Imadeldin E. Aradaib

**Affiliations:** 1 Molecular Biology Laboratory (MBL), Department of Clinical Medicine, Faculty of Veterinary medicine, University of Khartoum, P.O. Box 32, Khartoum North, Sudan; 2 Department of Microbiology, Faculty of Science, University of Kassala, Kassala, Sudan; 3 Department of Internal Medicine, Faculty of Science, University of Kassala, Kassala, Sudan; 4 Department of Medical laboratory Sciences, Faculty of Medicine, University of Elgadarif, Elgadarif, Sudan; 5 Deanship of Scientific Research, Alneelain University, Alneelain, Sudan; 6 Zamzam Unit for Medical Research (ZUMR), Vectore Borne and Zoonotic Diseases Research Laboratory, Zamzam University College, Khartoum, Sudan; 7 Deanship of Scientific Research, Moghtaribeen University of Africa, Khartoum, Sudan

**Keywords:** Epidemiology, Survey, Dengue virus, ELISA, Sudan

## Abstract

**Background:**

Acute arboviral infections are distributed worldwide including Sudan, and dengue fever (DENV) is not an exception. The virus activity has recently been frequently reported in Kassala State, eastern Sudan. However, an appropriate epidemiological study would be necessary to provide accurate and precise estimates of the magnitude of recent DENV transmission in this area of endemicity.

**Methods:**

In the present investigation, a cross sectional study was conducted to advance beyond the current knowledge of the epidemiology of the disease in Kassala State. The prevalence of the disease was estimated and associated risk factors were determined. Sampled sera were collected and screened for recent dengue transmissionas as determined by DENV-IgM enzyme-linked immunosorbent assay (ELISA). The collection of data for risk assessment was supported by a well designed structured questionnaire.

**R**esults**:**

The prevalence of recent DENV infection was estimated to be (11.42%). Potential risk factors to DENV seropsitivity include, age (OR = 3.24, CI = 1.81–5.77,*p*-value = 0.001); low income (OR = 3.75, CI = 1.57–8.93, p-value = 0.027); mosquito control (OR = 4.18, CI = 2.33–7.51, p-value = 0.004); and localities.

**Conclusion:**

The present study showed a high rate of circulating DENV IgM antibodies among the participants of the study (11.42%), suggesting recent transmission of DENV in Kassala State, eastern Sudan. The frequent occurrence of DENV infections necessitates the need for improved surveillance programs and prevention measures to combat this important arboviral disease in Sudan.

## Background

Dengue fever (DF) is caused by dengue virus (DENV), a positive-sense single stranded RNA virus of the genus *Flavivirus* in the family *Flaviviridae* [1, 2]. DF has been reported as one of the most important arboviral disease in many parts of the world including the Sudan [3–5]. DENV is spreading very rapidly resulting in emerging infections world-wide [6]. The high incidence of the disease has become of great concern to the public health officers world-wide [7–9]. In the recent years, DENV has spread all over the Sudan resulting in frequent occurrence of sporadic cases and multiple outbreaks [10–13]. The major economic losses caused by DENV infections in Sudan are almost exclusively confind to the Kassala state, eastern Sudan [10, 11, 14–16]. DENV activity usually varies from frequent sporadic cases to large explosive outbreaks. Clinical presentations of infected patients varies from mild fever to involvement of the circulatory system resulting in hemmorhagic manufistation with subsequent development of a more severedengue hemorrhagic fever (DHF). The clinical hemmorahagic disease leads to substantial increase in vascular permeability, which leads to dengue shock syndrome (DSS) followed by death [17, 18]. It is well documented that four DENV serotypes (DENV-1, DENV-2, DENV-3 and DENV-4) are circulating globally. DENV serotypes 1, 2 and 3 were reported to be endemic in some parts of the country [19–21]. However, DENV-4 is yet to be reported in Sudan. Several epidemic cycles of dengue have been recorded in the eastern States including, the Red Sea and Kassala [10, 11, 14, 20]. In the last few years, DENV activity has also been documented in the western part of the Sudan including the States of Darfur and Kordufan. DENV-1 and DENV3 were associated with the disease outbreaks in these States [22, 23]. In 1986, an outbreak of acute febrile disease caused by DENV-1 and DENV-2 was reported for the first time the Red Sea State, Sudan. Very recently, we reported on an exceptionally high prevalence (47.6%) of DENV-specific IgG in El-Gadarif State, eastern Sudan, where the disease has never been recorded before [24]. The State of El-Gadarif has several agricultural schemes and is boardring Kassala State to the south west side. In Sudan, several seroepidemiological studies were conducted to evaluate previous DENV infections by detection of DENV IgG antibodies. However, only two studies were conducted to evaluate recent transmission of DENV in the locality of Kassala and not the whole State. A previous seroepidemiological survey for DENV IgM, using Panbio (DF IgG and IgM) ELISA kits, reported a very low prevalence of 0.6% among residents of Kassala locality [16]. However, the underestimated prevalence of DENV IgM was attributed to the limitation of the ELISA assay to accurately detect IgM in sera from the study participants. The fact that the survey was conducted in the low transmission season of the year has also contributed to the low recent DENV transmission in the State. An appropriate epidemiological study would be necessary to provide more precise estimates of the magnitude of recent DENV transmission. On the other hand, a very high prevalence of 71.7% was reported among febrile patients admitted to Kassala Hospital during an outbreak of the disease, 2010 [14]. The exceptionally high prevalence of DENV IgM (71.7%) could be justified as the study was conducted during disease outbreak among symptomatic participants. DENV is endemic in the Sudan and the virus is probably actively circulating throughout the year, with a peak incidence between September and November, which coincides with the high rainy season. The detection of DENV IgM/ IgG antibodies and subsequent recovery of the virus from infected patients have been reported in eastern regions of the Sudan [10, 11, 19–22]. Recently, high incidence of dengue fever has been reported among residence of Kassala state as witnessed by frequent DENVsporadic cases and occuasionalmultiple outbreaks [19, 25]. Therefore, improved surveillance systems should be implemented to obtain comprehensive information on the epidemiology of DENV in Sudan. It is worth mentioning that implementation of improved surveillanceshould facilitate prediction and detection of recent infections and subsequent understanding of the ecology and biology of the virus, and the molecular epidemiology of the disease in this area of endimicity. From a public health prospective, we believe an accurate estimation of prevalence of new cases of DENV infections and identification of the associated risk factors are urgently needed. In the present investigation, serum samples were collected during the transmission season of DENV. In addition, participants from all localities of Kassala state were included in the study to provide a more accurate estimate of recent transmission of DENV. We anticipated that this study would be expected to provide a reliable and accurate epidemiological data, which assists in facilitating the control of the disease and prevent an expected DENV outbreak among residents of Kassala State, Sudan.

## Methods

### Study area

**Fig. 1:**
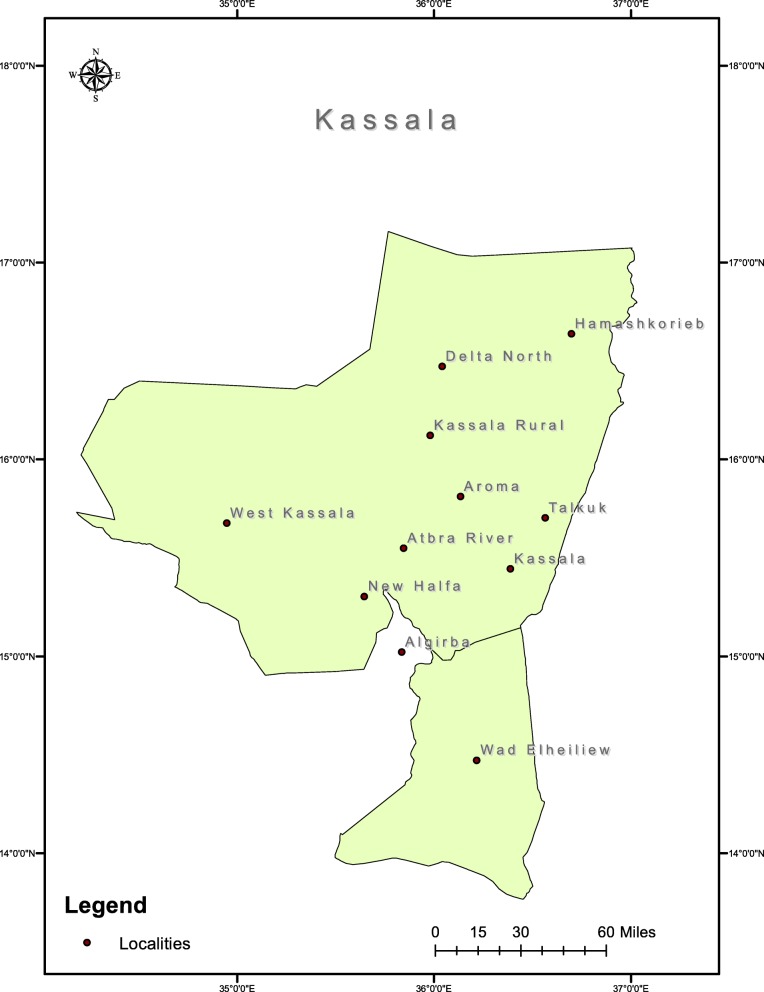
A map showing All localities included in the study area of Kassala State, Sudan

The present cross-sectional study was conducted in Kassala State, one of the poorest regions in Sudan, during the period between August, 2017 and May, 2018. Refugees and internally displaced population are hosted in this state. Most of the populations in the rural areas suffer of acute poverty and limited development prospects. The residentmigrated mainly from the war and rural areas affected by drought and desertification where the environmental conditions have continued to deteriorate over the years. The state experienced poverty and food insecurity that affect the region in the recent years. The state has an area of 36,710 km^2^ and an estimated population of approximately 1,400,000. Kassalastate is bordered by Eritrea and Ethiopia to the east, the Red Sea state to the north, Khartoum and the River Nile states to the west and the Gadarif state to the south west. The State is located between latitudes 15.8058° N and longitudes 35.5658° E in the semi-desert tropics. The climate is hot and rainy in summer but cold in winter. The fall extends from July to November, with an average 268 mm annual rainfall. The average annual temperature is 29.2 °C. Kassala state is composed of 11 localities, which include Kassala, Rural Kassala, West Kassala, New Halfa, Atbara River, Hamashkorieb, Elgirba, Delta North, Aroma, Talkuk, and Wad Elheiliew as illustrated in Fig. 1.

**Table 1:** Univariate analysis for the association between potential risk factors and DENV infection among residents of Kassala State, Sudan, using chi-square test (χ2)

Risk factors	Cases tested	Cases affected (%)	df	*χ*2	*p*-value
Locality			10	19.37	0.044
Kassala	105	8 (7.6%)			
R. Kassala	84	11 (13.1%)			
W. Kassala	70	12 (17.1%)			
New Halfa	83	7 (8.4%)			
Atbara River	70	9 (12.9%)			
Hamashkorieb	53	6 (11.3%)			
Elgirba	49	7 (14.3%)			
Delta North	50	1 (2.0%)			
R. Aroma	22	(00.0%)			
Talkuk	61	7 (11.5%)			
Wad Elheiliew	54	12 (22.2%)			
Age			1	17.06	0.001
young	309	18 (5.87%)			
Old	392	62 (15.8%)			
gender			1	1.16	0.281
female	320	32 (10.6%)			
male	381	48 (12.6%)			
Education			4	1.13	0.897
illiterate	177	19 (10.7%)			
primary	233	30 (12.9%)			
secondary	146	14 (9.6%)			
university	92	11 (12.%)			
informal study	53	6 (11.3%)			
Income			2	3.98	0.136
low	440	56 (12.7%)			
medium	146	17 (11.6%)			
high	115	7 (6.1)			
Work			1	0.05	0.809
unemployed	298	33 (11.1%)			
employed	403	47 (11.7%)			
Disease awareness			1	3.38	0.06
no	645	75 (12.3%)			
yes	56	5 (5.6%)			
Mosquito net use			1	6.16	0.013
no	342	43 (12.3%)			
yes	359	37 (10.6%)			
Mosquito control			1	12.38	0.001
No	313	126 (40.3%)			
yes	388	205 (53.6%)

**Table 2:** Multivariate analysis, using logistic regression model, for significant association (p < 0.05) between risk factors and dengue fever infections among residents of Kassala State, Sudan

Risk factors	OR	95%C I	*P*-Value
Age			
Young	Ref		
Old	3.24	1.81–5.77	0.001
Income			
high	Ref		
low	3.75	1.57–8.93	0.027
Mosquito control			
yes	Ref		
no	4.18	2.33–7.51	0.004
Locality			
Aroma	Ref		
W. Kassala	2.81	1.07–7.88	0.045
Wad Elheliew	3.07	1.08–8.36	0.030

### Study design, specimen collection and preparation

The blood samples were collected during the months of August to November, 2017. High infection rates occur during these months of the year, which coincise with the rainy season. Recent DENV transmission was determined as monitored by detection of DENV IgM antibodies. A cross sectional study was conducted to estimate the incidence rate of recent DENV-infections as judgedby detection of the specific IgM antibodies and to determine the potential risk factors associated with the disease. The participants for the study were selected from all 11 localities of Kassala State using a multi-stage cluster sampling technique was used to select the study participants [26]. In the first stage, the clusters, which were equivalent to a Popular Administrative Units were selected using the probability proportionate. In the second stage, households were selected using a systematic random sampling technique. Details of the selection procedure were similar to that described in a previous report [24]. Blood samples were collected from the participants, and sera were separated and kept frozen at − 20 °C until used for the detection of DENV-specific IgM antibodies by the enzyme-Linked Immunosorbent Assay (ELISA).

### Questionnaire

All participants from the above 11 localitie, targeted in this study, were subjected to interviews to obtain some information related to the risk assessment. A well-designed structured questionnaire was employed for this purpose. The questionnaire, covers socio-demographic characteristics including; age (young age, > 5 and < 18 years-old; old age > 18 year-old); gender (male and female); disease awareness (yes, no); work (employed, unemployed); education (illiterate, primary, secondary, University); household income which includes, low income (less than 2000 Sudanese Ginah (SDG), medium income (more than 2000 but less than 3000 SDG) and high income (more than 3000 SDG); mosquito net use (present or absent), and mosquito control (practiced or not practiced). The mosquito control includes the use of insecticides and mosquito nets. A Supplementary copy of the questionnaire is attached to the manuscript as a an additional file for clarity of the study (Additional file 1).

### Sample size

In a previous report the prevalence of DEMV-IgM seropositivity, during disease outbreak in Kassala State, was reported to be as high as 71.7% among patient admitted to Kassala hospital [14]. The second report showed a very low prevalence of (0.6%) as the study involved participants during dry season of the year where vector transmission is very rare [16]. The two seroepidemiological surveys were conducted in the locality of Kassala. None of the 2 studies involved participants from all localities of the State. In the present study, representative serum samples were obtained from participants of all 11 localies of Kassala. In addition, the serum samples were collected during the transmission season of DENV to obtain an accurate estimation of recent transmission. Because of the high variation between the two studies, we assumed the DENV-IgM seroprevalence to be 50%. The required sample size at 95% confidence and 50% prevalence and 0.04 absolute precision was calculated to be 600. A design effect of 2 and a non-response rate of 10% were considered to adjust for the sampling technique. The formula for the calculation of the sample size was applied as described by Dean et al. [27]. study participants were selected from all the 11 localities of Kassala State of DENV.

### Study inclusion and exclusion criteria

Any individual of both gender aged ≥18 years-old was included in this study. The samples were collected from symptomatic population. The symptoms include fever and rash for the last 5 days with or without hemorrhagic manifestations, vomiting, headache, and joint pain. Participants who are not residing in Kassala state for the previous 6 months including visitors and travellers were excluded from the study.

### Ethics approval and consent to participate

The ethical clearance for this study was kindly provided by the Ethics committee, Kassala State Ministry of Health, Sudan. Written consent for the purpose of this study was obtained from all participants and the objectives of the study were made clear prior to obtaining the informed consent.

### Enzyme linked Immunosorbent assay

The ELISA assay was performed using a commercially available IgM DENV ELISA Kit (Euroimmun AG, Luebeck, Germany), in accordance with the manufacturer’s specifications. The test samples were considered positive if the optical density was > 50% of the mean of the negative controls.

### Statistical analyses

The data were double checked before entry into the computer. Univariable analysis using Chi-square (χ2) test and multivariable analysis using logistic regression were calculated. Final results for risk factors were tabulated as odd ratios (OR) with 95% confidence intervals (C.I). Significant association between the dengue fever seropositivity and associated risk factorswhere considered when *p* < 0.05 is obtained. Statistical analysis using SPSSStatistical packageversion 21.0) was applied to determine thee prevalence and the potential risk factors. Details of statistical analysis were described else where by Eldigail et al. [24].

## Results

DENV-specific IgM ELISA assay was employed for detection of early antibody response to recent infection. Serum samples from al1 participants were tested in duplicates for the presence of DENV infection. Recent infection to DENV, as determined by DENV-specific IgMantibodies, was detected in 80 (11.42%) out of 701 participants using the ELISA assay. The highest rate of DENV seropositivity was recorded in the locality of Wad Elheiliew (22.2%) whereas the lowest rate was reported in Delta North (2.0%). However, Rural Aroma was found to be free of recent DENV infection (0.00%). Initially, the univariate analysis indicated that six risk factors with p- value < 0.25 (two tailed; α = 0.25) were significantly associated with DENV-antibodies as calculated in the χ2 test. These risk factors included locality (p- value = 0.044), age (*p*- value = 0.001), income (*p*- value =0.136), disease awareness (*p*- value = 0.06), mosquito net use (*p*- value = 0.013), and mosquito control practice (*p*- value = 0.001). The results of the univariate analysis are presented in (Table 1). Final model of multivariate analysis using logistic regression was applied to exclude confounding factors, which could be encountered in the initial results of univariate analysis. The final results using logistic regression indicated that only four potential independent risk factors were found to be significantly associated with DENV infection. These potential risk factors included, age (OR = 3.24, CI = 1.81–5.77, *p*-value = 0.001); low income (OR = 3.75, CI = 1.57–8.93, p-value = 0.027); mosquito control (OR = 4.18, CI = 2.33–7.51, p-value = 0.004); and localities. The two localities associated with high DENV serpositivity included West Kassala (OR = 2.81, CI: 1.07–7.88, *p* value = 0.045) and Wadelheliew (OR = 3.07, CI: 1.08–8.36, *p*-value = 0.03). The results of the significant association between DENV seropositivity and potential riskfactors in the final model are presented in (Table 2).

## Discussion

Dengue virus (DENV) is a single stranded RNA arbovirus of the geus *Flaviviru*s in the family *Flaviviridae.* DENV causes an acute febrile illness, which may develop in clinical hemorrhagic manifestation followed by shock [2]. The surge of construction of new cities along the River Nile and the Red Sea has let to urbanization in different parts of Sudan. In addition to urbanization, climate changes and increased human movements have contributed significantly to the global spread of DENV [4, 5, 9, 28]. The prevalence rate of DENV seropositivity in Kordufan and Darfur State of western Sudan were reported to be 27.7 and 15.7%, respectively [22, 23]. Interestingly, the present study showed a high prevalence of IgM antibodies of recent DENV infection among the residents of Kassala State (11.42%), suggesting recent transmission of DENV during the period of the study. Older participants are at 3 times higher at risk compare to younger age group (OR = 3.24, CI = 1.81–5.77, *p*-value = 0.001). Most of the participants exposed to recent dengue fever are over 18 years old. In addition, the present study indicated that DENV seropositivity increased among low-income participants. Low income residents were almost 4 times higher at risk compared to high income residents (OR = 3.75, CI = 1.57–8.93, *p*-value = 0.027). A considerable number of low income residents conistitutes refugees and internally displaced population. Most of the residents in Kassala State suffer acute poverty and limited development prospects. In addition, the State also experienced food insecurity, which was reflected very badly on the residents welfare in the recent years. The majority of the populations work in private sectors with minimum payment and others are unemployed. This cituation resulted in poor socioeconomic status, which contributed to high seropositivity to DENV. It is suggested that poor socio-economic status of the population would result in contamination of the environment with infectious diseases and provide suitable habitat for breeding of mosquito vectors, *Aedes aegypti*. The present study also showed significant association beteen DENV seropositivity and mosquito control of the primary vector (OR = 4.18, CI = 2.33–7.51, *p*-value = 0.004). The leve of DENVseropositivity was significantly reduced by at least four times when the application of insecticides was practiced. It should also be noted that Kassala State has several agricultural schemes and irrigation projects, which favour the environment condition for endemicity of DENV. In addition, the State is usually affected by the annual floods of El-Gash regional river during the rainy season. These environmental conditions provides suitable habitat for the breeding of the mosquito vector. The control of dengue vector is necessary to prevent the spread of the virus and to reduce the critical impact of disease burden in Sudan and the African continent at large. Future entomological studies would be required to determine the distribution of the vector in Sudan to facilitate control and management of the disease. There was also positive association between DENV seropositivity and the localities of WestKassala (OR = 2.81, CI: 1.07–7.88, *p* value = 0.045); and WadElheliew (OR = 3.07, CI: 1.08–8.36 *p*-value = 0.03). This is probably due to high rain fall in these localities, which provide suitable habitat for the mosquito vector that transmits the disease. Residents of of Wad Elheliewlocality are at 3 times higher at risk of becoming infected with DENV compared to other localities, suggesting increased DENV endemicity of this locality. On the other hand, the locality of Rural Aroma was found to be free of recent DENV infection (0.00%). This could be attributed to the hot and dry weather, which does not provide a suitable habitat for the breeding of the mosquito vector. However, it should also be noted that the lowest number of samples was collected from participants of this locality. It should be taken into consideration that Kassala State shares boarder with Ethiopia and Eretria to the east side of Sudan. Increased human movement due to international trade has recently been observed in the region. It is, therefore, suggested that movementof DENV is likely to occur between these countries in east central Africa. How DENV travels is unclear but probably involves movement of infected mosquitoes via commercial trade or travel of viremic patients [8, 28–30]. It is also possible that DENV spreads to eastward across the Red Sea into Saudi Arabia through the winds. More sequencing data of DENV isolated in the region and subsequent phylogenetic analysis would be required to identify the DENV serotypes and to determine the virus genetic lieages circulating the region. In Sudan, there is no well-established active dengue surveillance system and physicians do not usually consider dengue during clinical presentation and differential diagnosis. Circulation of dengue virus serotype 2 was reported to be associated with disease outbreak in Kassala State, 2017 [17]. However, none of the remaining serotypes were reported in this State. More than one DENV serotypes are are belived to be co-existing in the State. Further study would be required to confirm this assumotion. Severe cases of dengue hemorrhagic fever are usually associated with the presence of multiple serotypes of DENV in areas of endemicity [17, 18, 31]. Therefore, further studies are needed to provide some information on virological aspect and molecular biological detail of DENV in Kassala State. The study suggested that an established diagnostic facility and appropriate control program should be considered as a top priorityto combat this important arboviral infection [32, 33].

### Limitation of the study

The present study provides important information regarding the prevalence and risk factors of recent transmission of DENV in Kassala State, Sudan. Despite the fact that this is the first report on sero-prevalence of dengue in all localities representing Kassala State, during the transmission season of the disease, some limitations were recorded for careful consideration in the future. One of the limitations of the study is that the seropositivity was assessed by detecting DENV IgM antibody levels. It is well documented that detection of IgM is useful in an epidemiological survey to identify recent infections. However, the ELISA IgM antibodies can cross-react with other flaviviruses. Members of flavivirus serogroup, other than DENV, have not been reported in eastern part of Sudan including Kassala State. At the same token, negative results will not exclude the disease, as the production of DENV IgM requires at least 1 week window period post infection. We recognized that DEN-NS1 ELISA assay is useful to eliminate the possibility of cross reaction with related flaviviruses. However, DENV-NS1 ELISA can detect positive cases at about the first 2 weeks of infection. Serum samples collect at a later stage are likely to be missed during the performance of the assay resulting in false negative results. Therefore, it should be noted that employment of different assays for screening of recent DENV transmission would change the interpretation of the results substantially given their different window of detection. Virus serum neutralization test would be more appropriate to identify DENV serotypes and to eliminate the possibility of cross reaction. In addition, virus isolation attempts and subsequent molecular characterization studies were not conducted in this study. Future studies should consider the detection and identification of circulating DENV serotypes and associated genotypes in this region of the African continent. Further investigations should be conducted in the future to overcome the limitations of the study.

## Conclusions

Our study illustrates recent DENV transmission during the rainy season of the year resulting in high prevalence of 11.42% in Kassala State of eastern Sudan. Age, low income, mosquito control and localities showed significant association with DENV IgM seropositivity. It is suggested that, virus isolation attempts should be conducted to identify DENV serotypes and associated genotypes circulating in Sudan. In addition, generation of whole viral genome sequences and subsequent phylogenetic relatedness would be advantageous in tracing the movement of the virus in this region of the African continent. Differentia diagnosis for DENV should be considered when clinical presentation is conducted in a patient with symptoms indicative of acute febrile illness. The frequent occurrence of recent DENV infections necessitates the need for improved surveillance programs and prevention measures to combat this important arboviral disease in Kassala State, Sudan.

## Additional File

**Additional file 1.** A Supplementary copy of the questionnaire is attached to the manuscript as a an additional file for clarity of the study (Additional file. 1) showing the data employed for the identification of the risk factors associated with Recent transmission of dengue virus among residents of Kassala State, Eastern Sudan.

## Data Availability

Data and materials are available upon request from the corresponding author.

## Abbreviations

DEN: Dengue
DEN-V: Dengue virus
Ig G: Immunoglobulin G
Ig M: Immunoglobulin M
ELISA: Enzyme-linked immunosorbent assay
RT-PCR: Reverse transcriptase polymerase chain reaction
Μl: Microliter
CI: Confidence interval
OR: Odd ratio
HRP: Horse radish peroxidase
