# Supplementary material for: Recent transmission of dengue virus and associated risk Facors among residents of Kassala state, eastern Sudan
Source: BMC Public Health. 2020 Apr 19;20:530. doi: 10.1186/s12889-020-08656-y (PMC7168835; doi:10.1186/s12889-020-08656-y)
Supplement: Supplementary file 1 — Additional file 1. A Supplementary copy of the questionnaire is attached to the manuscript as a an additional file for clarity of the study (Additional file. 1) showing the data employed for the identification of the risk factors associated with Recent transmission of dengue virus among residents of Kassala State, Eastern Sudan. [file 12889_2020_8656_MOESM1_ESM.docx]

**Questionnaire**

Recent Transmission of Dengue Virus and Asssociated Risk Factors Among Residents of Kassala State, Eastern Sudan.

**Name………………………………………………………**

**Locality……………………………… ……………..**

**Address…………………………………………..**

**Phone number…………………………………….**

**1-Ag**

1. Young age: > 5 years-old and < 18 years-old
2. Old age > 18 years-old

**2-Gender**

1. Male
2. Female

**3-Marital status**

1. Married
2. single
3. divorce

**4-Education**

1. Literate
2. illiterate
3. primary school
4. Secondary school
5. University

**5-Income**

500-100 Sudanese pounds

1001-3000 Sudanese pounds

>3000 Sudanese pounds

**6. Awareness of dengue**

**Yes No**

**7. Use of mosquito nets**

**Yes No**

**8. application of mosquito control**

**Yes No**
